# Fiber Optic Sensors for Structural Health Monitoring of Air Platforms

**DOI:** 10.3390/s110403687

**Published:** 2011-03-25

**Authors:** Honglei Guo, Gaozhi Xiao, Nezih Mrad, Jianping Yao

**Affiliations:** 1 Microwave Photonics Research Laboratory, School of Information Technology and Engineering, University of Ottawa, Ottawa, ON K1N 6N5, Canada; E-Mails: hguo062@uottawa.ca (H.G.); jpyao@site.uottawa.ca (J.Y.); 2 Institute for Microstructural Sciences, National Research Council Canada, Ottawa, ON K1A 0R6, Canada; 3 Air Vehicles Research Section, Defence R&D Canada, Department of National Defence, National Defence Headquarters, Ottawa, ON K1A 0K2, Canada

**Keywords:** aircraft, damage detection, fiber Bragg grating, fiber optic acoustic sensor, load monitoring, structural health monitoring

## Abstract

Aircraft operators are faced with increasing requirements to extend the service life of air platforms beyond their designed life cycles, resulting in heavy maintenance and inspection burdens as well as economic pressure. Structural health monitoring (SHM) based on advanced sensor technology is potentially a cost-effective approach to meet operational requirements, and to reduce maintenance costs. Fiber optic sensor technology is being developed to provide existing and future aircrafts with SHM capability due to its unique superior characteristics. This review paper covers the aerospace SHM requirements and an overview of the fiber optic sensor technologies. In particular, fiber Bragg grating (FBG) sensor technology is evaluated as the most promising tool for load monitoring and damage detection, the two critical SHM aspects of air platforms. At last, recommendations on the implementation and integration of FBG sensors into an SHM system are provided.

## 1. Introduction

It is well known that a significant number of military aircraft fleets around the world are operating beyond their design life cycle. In Canada, the CF-188 fleet that was expected to be replaced in 2004 is still in operation and is now expected to be taken out of service in 2018 [1]. In Australia, the F-111C fleet is expected to be in service 20 years beyond its designed life cycle. In the United States, like in other countries, life extension programs are in place to extend the life cycle of several aircraft fleet including the F-4 and B-52 [2]. To continue to meet airworthiness and availability requirements, aggressive inspection and maintenance regimes are expected to be imposed, resulting in added costs of maintenance and support. Although conventional schedule-based inspections contribute greatly to the safety and reliability of these platforms, they are also the main contributors to the high operation costs [3]. Additionally, these periodic on-ground inspections might require the disassembly and reassembly of inspected components, further increasing the potential for introducing damage and degradation of structures and auxiliary systems, such as electrical wiring and hydraulic lines [4]. The concept of structural health monitoring (SHM) stands to reduce the complexity and the costs associated with these traditional approaches, and the exploitation and implementation of SHM tools are expected to replace schedule-based inspections by the on-board and real-time monitoring to reduce platform life cycle cost, improve safety and reliability, and extend operational life cycle [5].

A wide range of embedded and attached sensors have been studied for SHM applications, including strain gauges, accelerometers, fiber optic sensors, active ultrasonic sensors, passive acoustic sensors, wireless sensors, *etc*. Among them, fiber optic sensor has been emerging as an increasingly important tool for SHM due to their unique advantages in sensitivity and multiplexing capability. Aircraft SHM generally consists of two critical aspects, *i.e.*, operational load monitoring and impact damage detection [4]. For load monitoring, strain gauges, accelerometer and fiber optic sensors are the main choices. Both strain gauges and accelerometer are relatively mature, but their wirings pose significant challenges for the sensor deployment. On the other hand, one optic fiber can be used to multiplex tens or hundreds of fiber optic load sensors, thus greatly lessening the wiring issue. For damage detection, ultrasonic/acoustic sensors based on piezoelectric materials are currently the standard pick even though they are still in the development stage. Similar to strain gauges and accelerometer, the multiplexing capability of these piezoelectric sensors is limited and their wiring is also a big challenge for the deployment. Again using fiber optic acoustic sensors can effectively address this wiring issue.

Over several decades, a wide range of fiber optic sensor approaches have been intensively studied [5] and widely implemented [6]. Among these advanced candidates for the development of structural health monitoring systems, fiber Bragg gratings (FBG) have received the wider visibility and acceptance in both R&D and field applications [7]. The recent trends in FBG-sensor based structural health monitoring for aircraft structures include the *in-situ* detection of structural strain, the hybrid use of FBG sensors and acoustic inspections designed for the damage detection inside the structures, and the highly multiplexed FBG sensor system for other structural related parameters detection.

This paper will firstly give brief introduction for the two aspects of the aircraft SHM, the operational load monitoring and damage detection. And then, an overview of fiber optic sensor technology will be provided. Specifically, different types of fiber optic sensors will be evaluated for applications in the aircraft load monitoring (mainly strain measurement), and FBG sensors for aircraft damage detection will be discussed. Finally, recommendations of an FBG sensor based aircraft SHM system for the monitoring of both operational load and damage are proposed.

## 2. Structural Health Monitoring of Air Platforms

As mentioned above, load monitoring and damage detection/monitoring are the two critical aspects of structural health monitoring for air platforms. Operational load monitoring is used to support the fatigue life management, such as estimating the pattern of structural fatigue life and providing information about the possible structural damage. More specifically, the fatigue life assessment is evaluated by measuring the local stresses, which can be either directly obtained using sensors or derived from various global flight parameters and structural usage data [8,9], such as flight speed, altitude, mass, and acceleration. The latter approach is regarded as an unreliable method since loads need to be converted from other parameters and fatigue life calculations are based on imprecise damage accumulation rules. Nevertheless, it is the only option until the emerging of effective and reliable strain sensors. Currently, load monitoring is performed by the combination of the two methods, *i.e.*, limited number of strain sensors mounted in some critical points for direct measurement and monitoring of flying parameters for the estimation of loads in other locations. Obviously, the number of those critical points selected will have a significant impact on the final definition of the load monitoring.

Electrical strain gauges are currently the most mature technology for load monitoring. However, each strain gauge needs its own dedicated wires. This makes the deployment of multiple strain gauges difficult due to the wiring challenges. As a result, the number of points being monitored is generally limited from 5 to 20. Most end-users would like to see more points being monitored if this would not increase the system complexity and operation cost. An alternative method is to use fiber optic strain sensors. Besides high performances, a large quantity of fiber optic sensors can be easily multiplexed in a single optical fiber and potentially monitored remotely, thus greatly lessening the wiring issue.

As the only proven aircraft SHM method, operational load monitoring has already been applied to different types of military and civilian aircrafts for the estimation of the accumulated fatigue damages and the remaining aircraft operation life. Nevertheless, load monitoring alone is not able to directly detect and monitor the structural damages (another critical aspect of SHM of air platforms). For example, operational load monitoring can not provide direct information of damages resulting from corrosion in metallic or debonding/delamination in composite materials. Therefore, another method capable of directly detecting/monitoring the damages is required. This can be potentially achieved by integrating sensing system onto or into the structural components, thus allowing the direct measurement of the occurrence, size, and location of damages. Currently the only proven method for damage detection is ultrasonic/acoustic non-destructive technology, even though the technology itself is still in the development stage. When using this method, aircrafts have to stop service and be manually checked point by point or by complicated instruments mounted on a moving cart. Furthermore, some aircraft structures might need to be dissembled for the inspection, which could cause unpredicted damages to the structures being inspected. Due to the complexity of this inspection, highly experienced and educated technicians are needed for the work. From the viewpoint of the method itself, ultrasonic detection is based on an actuation-sensing process, where the actuators and sensors are made of piezoelectric (PZT) materials. Present ultrasonic sensors are bulky as each of them has two wire leads for picking up signals and these wires require heavy shields to avoid adverse interference such as electromagnetic interference (EMI) and radio frequency interference (RFI). They also need a pre-amplifier for the signal amplification, adding two more wires and extra cost. In addition, such sensors suffer from poor multiplexing capability just as the strain gauges. A potential solution to the damage detection for air platforms is to use fiber optic sensors. They can not only pick up acoustic signals just like the conventional PZT sensors, but also be multiplexed in one optical fiber and monitored remotely.

What really counts for fiber optic sensors for SHM is the possibility of realizing a fused system with multifunctional measurement capability in contrast to those conventional sensors. Specifically, fiber optic sensors can perform static strain measurement in a large scale (thousands of μstrains) at a low speed (200 Hz) for potential operational load monitoring and ultrafast strain measurement in a small scale (tens of μstrains) at an ultra-fast speed (500 kHz) for potential damage detection. This would significantly reduce the complexity of SHM systems and the costs related to SHM.

## 3. Overview of Fiber Optic Sensor Technologies

**Figure 1.**
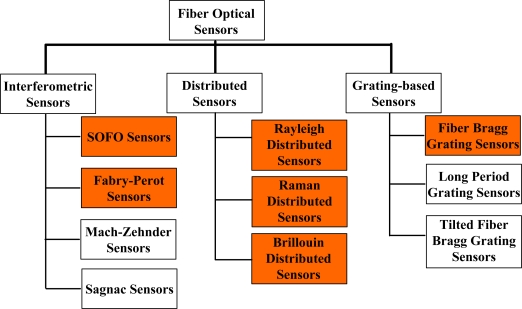
An overview of fiber optic sensor technologies.

To date, several fiber optic sensor technologies have been developed and some of them are commercially available [5]. In general, they can be categorized into three classes: interferometric sensors [10,11], distributed sensors [12–14], and grating-based sensors [15–17]. Each category has a large variety targeting diverse types of measurements and applications. Details are shown in Figure 1. The highlighted sensor technologies in Figure 1 represent that they have already reached an industrial level with commercial products employed in real industrial applications. Their performances are summarized in Table 1.

### 3.1. Interferometric Sensors

An interferometric sensor is created by an intrinsic or extrinsic interferometric cavity along an optical path [10]. Physical changes in structures are reflected by the changes of the optical phase difference between two interference light waves. The nature of interference renders the interferometric sensors the capability of measuring very small mechanical deformations bettering than µstrain (µɛ), *i.e.*, one part per million elongation or contraction. Well known and practical interferometric sensors include Fabry-Perot interferometric sensors and low coherent interferometric sensors (SOFO interferometric sensors) [11].

A Fabry-Perot interferometric sensor could have a resolution as high as 0.15 µɛ, a strain measurement range of ±1,000 µɛ with the capability of being extended to ±5,000 µɛ, and can be operated at temperatures ranging from −40 °C to +250 °C. Fabry-Perot interferometric sensors are very compact with a length from 1 mm to 20 mm and can be embedded into certain structural components without any weight penalty and adverse effects. However, they are suffering from the disadvantage of low multiplexing capability.

SOFO interferometric sensors are the most successful low coherent interferometric sensors for SHM, which have been reported being successfully deployed in more than hundreds of structures so far, including bridges, buildings, oil pipes and tunnels. In contrast with Fabry-Perot interferometric sensors, SOFO interferometric sensors are long-gauge sensors. They have a measurement range starting from 0.25 m to 10 m or even up to 100 m with a resolution in the level of micrometer with features of temperature insensitivity, high precision and stability. However, they are only suitable for the measurement of elongations and contractions at a low speed (0.1 Hz–1 Hz) and not capable of detecting the impact damages in aircraft structures.

### 3.2. Distributed Sensors

There are three types of fiber optic distributed sensors: optical time-domain reflectometry (OTDR) based on Rayleigh scattering, Raman optical time-domain reflectometry (ROTDR) based on Raman scattering, and Brillouin optical time-domain reflectometry (BOTDR) based on Brillouin scattering.

OTDR is the first generation of fiber optic distributed sensors, where Rayleigh scattering is used to reflect the attenuation profiles of long-range optical fiber links [12]. An optical pulse is introduced into an optical fiber link and the power of the Rayleigh backscattered light is measured by a photodetector as the light pulse propagates along the fiber link, which is normally used to determine the fiber loss, break locations, and evaluate splices and connectors.

ROTDR and BOTDR are employed for distributed sensing applications over the past few years. Their operation mechanisms are based on the nonlinearities of optical fibers, where additional spectral components are generated. These additional spectral components are affected by external environmental parameters. Thus, evaluating the spectral content in an appropriate way can determine the changes in external measurands.

ROTDR is based on the Raman-scattering phenomena, where both anti-Stokes components and Stokes components are generated [13]. The intensity ratio between these two components can provide temperature information at any given point along the fiber link as the fiber link itself is the sensing medium. Since the amplitude of the Stokes components is not dependent on temperature, ROTDR is only capable of measuring the temperature rather than the strain with a temperature resolution of 0.2 °C. The sensing distance of ROTDR is normally limited to approximately 8 km with a spatial resolution of 1 m.

In BOTDR, light launched into an optical fiber link is partially scattered back based on Brillouin-scattering phenomena [14]. The frequency of the scattered light is dependent on temperature and strain applied on the fiber link, which allows BOTDR to measure both temperature and strain. The standard BOTDR offers a measurement distance of 30 km and can be expanded up to 200 km. The spatial resolution is from 1 m to 4 m.

### 3.3. Grating-Based Sensors

**Figure 2.**
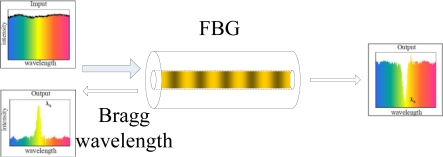
Functional principle of an FBG.

Fiber Bragg grating (FBG) sensors are regarded as the most mature grating-based sensors and have already been widely used [15]. An FBG sensor reflects a portion of the incoming light of a particular wavelength, called Bragg wavelength, and leaves the rest of the incoming light pass without altering its property as shown in Figure 2. The Bragg wavelength is defined by the fiber refractive index and grating pitch, which are affected by the external environment changes, such as temperature, strain, vibration and other parameters. All these changes are reflected on the Bragg wavelength shift. Therefore, by monitoring the Bragg wavelength shift, several measurands can be monitored using FBG sensors. In the past 20 years, wavelength multiplexing technology has been mature, hundreds (if not thousands) of wavelengths can be multiplexed in one single optic fiber. Current technology makes it possible to multiplex tens or hundreds of FBG strain sensors in one optic fiber and monitor them remotely. With the rapid development in the past few years, FBG sensors have been targeted as the major leading technology in contrast to other competing fiber optic sensor technologies. Besides its wavelength multiplexing capability, FBG sensors have the advantages of low cost, compact size, and good linearity. The grating length is usually in the order of 10 mm. The resolution is dependent on the wavelength interrogator, which is currently up to 1 pm, corresponding to 1 µɛ for strain measurement and 0.1 °C for temperature sensing.

In addition to the wide applications in temperature and strain sensing, FBG sensors have found applications in the measurement of acoustic/ultrasonic signals [16,17]. In other words, they can be used for damage and crack monitoring. In this type of applications, FBG sensors substitute the conventional PZT sensors to pick up the ultrasonic/acoustic waves to reduce the complicated wiring issue. FBG sensors have the potential to be deployed in a large quantity by utilizing their multiplexing capability, therefore, to achieve a full-scale monitoring. This feature is one of the most competitive advantages of FBG sensors over traditional PZT sensors and other types of fiber optic sensors. Principle and selected development of FBG acoustic sensor systems will be described in the next section of this review paper.

## 4. FBG Acoustic Sensors

Applications of FBG sensors have been well documented in several review papers [15,18–22]. These reviews primarily cover the applications of FBG sensors for the strain and temperature measurements in civil structures [20–22]. The development of an SHM system capable of monitoring both operational load and impact damage is of particularly advantageous for aircrafts. As discussed above, FBG sensors have shown this capability. In operational load measurement, strain sequence is required to deduce the load history and further estimate the fatigue life [23]. Systems based on FBG sensors for operational load monitoring are well established, developed and tested [24,25]. Industrial leaders in FBG sensors have published commercial solutions, such as a 17-FBG-sensor system developed by Micron Optics for monitoring operational load and temperature in a personal aircraft [26] and an FBG sensor based SHM system with a sampling rate in excess of 8 kHz developed by Insensys (UK) [in collaboration with Bell Helicopters and United Technologies Research Laboratories (UTRC)]. For the damage detection/monitoring, FBG sensor based system is still at its early stage [27,28], since this technique is based on acoustic and ultrasonic non-destructive evaluation (NDE) techniques and these two techniques are still under development. However, the emerging demands on directly monitoring of the crack existence and growth have accelerated the development of the acoustic and ultrasonic NDE techniques as they are the only proven methods.

### 4.1. Fundamental Principle

Lamb wave is one of the guided acoustic waves with its propagation vectors parallel to the structure surface. In addition, lamb wave could also couple its energy throughout the structure thickness. These two characteristics make lamb wave an ideal candidate for the SHM applications [27]. The detection of lamb wave using FBG sensors will be the subject of this review paper.

The interaction between lamb waves and FBG sensors is simple. Propagating lamb waves change the grating pitch of the sensor which then causes the Bragg wavelength to shift. By monitoring the Bragg wavelength shifts, lamb waves could be reconstructed. The structural information, such as the existence, the size, the location and the growth of cracks, can be obtained by analyzing the received lamb waves. Theoretical analysis for the response of FBG sensors to longitudinal and transverse waves are reported in [29,30] respectively. Though there are currently no standards to follow in determining the specifications of the FBG acoustic sensor system, it should be noted that only uniform strain applied on FBG sensors can be used to reflect the structural status. If the strain applied on FBG sensors are not uniform, signals from FBG sensors will consist of both wavelength shifts and spectrum broadening, which are very difficult to be interrogated and make it impossible to recover the lamb waves [31].

In order to meet the above condition, the grating length of FBG sensors should be less than the wavelength of the propagating lamb wave. In other words, the ratio between the wavelength of the lamb wave and the grating length of the FBG sensor needs to be considered. A numerical analysis on this ratio is detailed in [32] with the conclusion that only if the ratio exceeds a certain value, the lamb waves could be characterized by FBG sensors. An experiment in testing the ultrasonic responses of FBG sensors with different grating length is reported in [33]. In this test, an ultrasound signal at 2.25 MHz is applied as the guided signals and FBG sensors with grating lengths of 1-mm and 12-mm are used to pick up the ultrasonic signals respectively. Results show that the signal-to-noise ratios of the responses detected with the two FBG sensors are estimated to be 55 and 29 dB for the 1-mm FBG and 12-mm FBG respectively. It is seen that high sensitivity in ultrasonic signal measurement is achieved by reducing the grating length of FBG sensors. An ideal value of this ratio is set as of 6:1 to allow the response of FBG sensors to be effectively independent of the lamb wave wavelength [31]. For more common applications, this value can be set within the range from 1 to 4 depending on the requirement of sensitivity [34]. There are two limits for setting this ratio, 3 dB (50%) and 0.2 dB (95%) with the corresponding ratio of 1.2 and 4 respectively. Assuming that the grating length is 1 mm, the detectable lamb wave wavelength is 1.2 mm for the 3 dB limit and 4 mm for the 0.2 dB limit, corresponding to approximately 5 MHz for the 3 dB limit and 1 MHz for the 0.2 dB limit as of the lamb wave frequencies. Current commercial FBG sensors usually have a grating length of 10 mm, corresponding to 0.8 MHz for the upper limit of the mapping lamb wave signal. As typical frequency value of lamb waves used for SHM starts from 50 kHz, an FBG acoustic sensor system could be set up using commercial FBG sensors. The use of commercial FBG sensors could avoid the fabrication of special FBG sensors and reduce the cost.

### 4.2. Overview

FBG sensors have been used for ultrasonic/acoustic signal measurements in several different fields [35–41], but their applications for damage detections are relatively new. Reference [34] reports the comparison between FBG sensors and conventional PZT transducers in acoustic signal monitoring for damage detection in structures. The experimental work is implemented on a rectangular 1 mm × 400 mm × 400 mm aluminium plate, where three PZT transducers and one FBG sensor are located at the four corners respectively. In this work two different driving frequencies of 260 kHz and 460 kHz are selected to generate the fundamental mode acoustic waves. The acoustic waves are produced in a tone burst format since this format is the most common approach for the analysis. The use of burst signals allows the measurement of the time needed for a signal traveling from the source to the receiver. Then, the distance between the two points and the travelling velocity could be obtained if either one is known as the condition. The first conclusion of this work is that the higher frequency gives an advantageous result due to the less interference of the waves on the plate and the ratio of damage size and wavelength is bigger, resulting in a higher sensitivity to damage. According to the propagation principle, acoustic waves are reflected at the boundary of the aluminum plate, making the waves contain multiple reflected waves. It is difficult to interpret all these waves if a more complex structure is applied. The second conclusion of this work is that only the first two packages within the signal should be considered if the acoustic wave data is analyzed independently of the receiver or the frequency used, since the later packages are tested to be the responses from several reflections. The third conclusion is that FBG sensors could be used to record the acoustic signals and the results show FBG sensors offer the same quality as the ones from the conventional PZT transducers. A more detailed description on the experimental setup, results and mathematics for the data processing is documented in [42].

**Figure 3.**
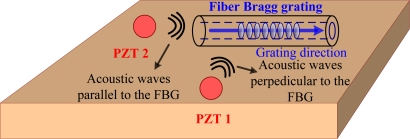
Configuration to characterize the directional property of an FBG sensor to acoustic waves.

Furthermore, the response of FBG sensors to acoustic waves is dependent on the relative positions of the signal source (PZT transducers) and FBG sensors, means that FBG sensors have high directivity [27]. In this case, the received signal amplitude is evaluated with respect to the directions of the incoming acoustic waves, which could be tested with the configuration shown in Figure 3.

The experiment is implemented in two steps. First, PZT 1 is activated and generates acoustic waves that are perpendicular to the FBG sensor. Second, PZT 2 is used to repeat the same procedures, where the acoustic waves are launched into the FBG sensor in a parallel case. Results show that the amplitude in the parallel case is 100-times stronger than the perpendicular case [27]. Both acoustic wave amplitude and direction can be calculated by introducing the relative amplitude into a suitable algorithm. This simple angular response of FBG sensors makes it possible to locate the acoustic waves. This is implemented by calculating the point of intersection of the directional vectors obtained from at least two FBG sensors.

A more sensitive FBG acoustic sensor configuration is proposed in [43], where a one-end-free and strain-free FBG sensor is packaged into a steel tube and the tube is bonded to a structure. Since the FBG sensor is isolated inside the tube, the acoustic waves do not directly interact with the FBG sensor. However, one end of the fiber is bonded to the structure and the acoustic waves are coupled into the fiber, travel through the fiber and reach the grating region. This configuration provides a more sensitive FBG acoustic sensor. Similar idea is also applied to implement an acoustic emission measurement [44].

In order to reduce the weight and fuel consumption, composite materials have been used to replace metal in aircraft structures [45]. FBG sensors fabricated on standard single mode fiber (125 μm diameter) might induce defects due to their relative large diameter when embedded into composite materials. To overcome this limitation, FBG sensors based on a small-diameter fiber are developed [46]. In this design, the diameter of the fiber is less than one-third of a standard single mode fiber. The sensors fabricated on this small-diameter optical fiber have been studied for the damage detections in composite structures [47–49].

Since active acoustic sensing can provide more flexibility than passive acoustic sensing, PZT transducers are needed to provide inspection acoustic waves, where FBG sensors are used as the receivers. In order to take the full advantages of fiber optics, it is desirable that the inspection acoustic waves could be generated using fiber optic techniques. For this, an all-fiber optic acoustic monitoring method is proposed and demonstrated [50]. In this technique, a portion of the fiber cladding is replaced by graphite-epoxy composite. This material is able to absorb the pulsed laser light and convert the energy absorbed to the rapid increase of local temperature. This then results in the rapid thermal expansion. With the thermal expansion of such cladding material, acoustic waves can be generated. In this design, both the acoustic wave source and receiver are based on fiber optics.

### 4.3. Wavelength Interrogation System

**Figure 4.**
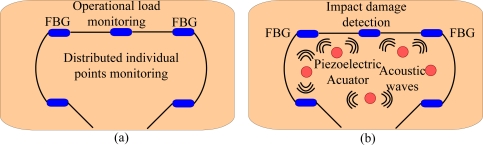
Two-dimensional structure monitoring. **(a)** operational load monitoring and **(b)** impact damage detection with hybrid use of FBG and acoustic actuators.

**Figure 5.**
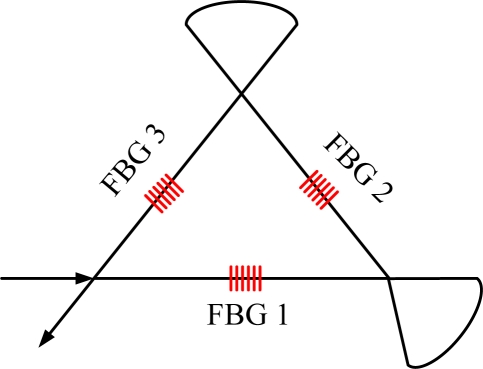
Rosette configuration of FBG sensors.

As discussed above, an FBG sensor array can be used for both operational load and damage detection. Illustration for integrating these two functions in a full-scale monitoring application is illustrated in Figure 4(a,b), respectively [51–53]. As it can be seen, tens of FBG sensors might be needed for this function integration. In addition, as an FBG sensor is sensitive to both strain and temperature, FBG temperature sensors are needed to perform the temperature compensation. Furthermore, in order to achieve accurate and reliable measurement, FBG sensors in a rosette configuration, as shown in Figure 5, are now widely applied for each sensing point [31]. Therefore, tens or hundreds FBG sensors are required in a FBG acoustic sensor system. This makes the interrogation very challenging.

Besides the requirement of interrogating tens/hundreds of FBG sensors simultaneously, interrogation speed from tens of kHz to several hundreds of kHz is required in order to pick up acoustic waves. Furthermore, strain changes induced by acoustic waves are very small. The amplitudes are at the level of tens of μɛ to several hundreds of μɛ [34]. Recall that the strain sensitivity of a typical FBG sensor with a Bragg wavelength of 1,550 nm is 1 pm/μɛ, therefore, high resolution in wavelength interrogation is also required for the measurement of acoustic waves.

**Figure 6.**
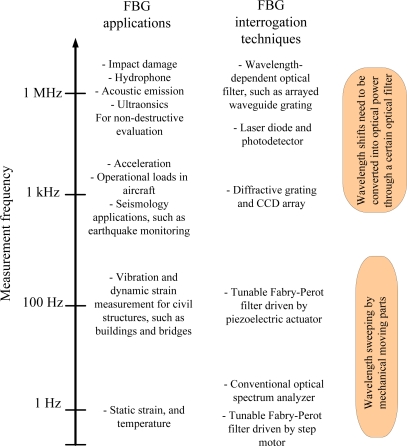
FBG interrogation methods classified by measurement frequency.

In recent years, high speed wavelength interrogation techniques have been developed with a speed over 100 kHz, such as a Fourier-domain mode-locked laser [54], wavelength-tunable mode-locked laser [55] and wavelength-swept laser based on fiber vibration [56]. However, these techniques have bulky size, high cost and complex configuration. Based on the description in [57], FBG interrogation methods can be classified by the measurement frequency as shown in Figure 6. As it can be seen, wavelength sweeping by mechanical moving parts, such as a tunable laser source or a tunable Fabry-Perot filter, is used for the wavelength interrogation with the measurement frequency under 1 kHz. For the measurement frequency over 1 kHz, Bragg wavelength shifts should be converted into optical power through a certain type of optical filter without introducing any mechanical moving parts.

**Figure 7.**
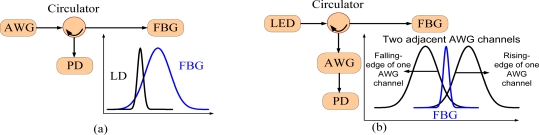
FBG interrogation system using. **(a)** laser diode (LD) and **(b)** AWG.

Several approaches have been developed for the wavelength interrogation in the acoustic wave measurement, including a matching FBG [58], a long period fiber grating [59], a laser diode [34], and an arrayed waveguide grating (AWG) [60]. Considering the reliability of the wavelength interrogation system, laser diode and AWG have superior features. The basic principle of the wavelength interrogation using a laser diode is simple. The wavelength of the laser diode is first set to a certain position of the FBG spectrum as shown in Figure 7(a). If the FBG spectrum shifts, the reflected optical power at the photodetector also changes, therefore, the wavelength shifts could be obtained from the changed optical power. The laser diode can be replaced with a tunable laser source to perform a dual operational load monitoring and damage detection [27]. In this case, the tunable laser source has two operation modes. One is to fix its output at a certain wavelength for the measurement of acoustic waves, which is the same as that of using a laser diode. The other operation mode is to enable the wavelength sweeping. In this case, its output laser can scan the spectrum for the measurement of operational load. The advantages of using a tunable laser source are the high resolution and accuracy due to its low noise and narrow linewidth laser output. However, it has limitations of high cost and difficulty in meeting the requirements to simultaneously interrogate multiple FBG sensors when measuring acoustic waves, since a tunable laser source has only one wavelength output each time.

Recent development of using an AWG as an interrogation unit has successfully addressed this limitation. An AWG is a key device in wavelength division multiplexed (WDM) optical communication systems, in which the AWG is used to implement wavelength multiplexing or de-multiplexing, to increase the transmission capacity of the communication system [61]. An AWG can also be used in a wavelength interrogation system [62–65]. The use of an AWG for acoustic wave measurement is to set up a linear wavelength-dependent optical filter as shown in Figure 7(b). The linear wavelength-dependent optical filter consists of two edges from two adjacent AWG channels, the rising-edge and the falling-edge. The Bragg wavelength is set at the middle of the two AWG channels. The power ratio of the two channels is dependent on the Bragg wavelength. Thus, by monitoring the optical power ratio, the information relative to the Bragg wavelength changes can be obtained [60–62]. Furthermore, the AWG spectrum could be tuned to obtain a high resolution and broad range in wavelength interrogation [63–65]. As a result, the tunable AWG interrogation system is able to measure the operational load. That means an AWG-based wavelength interrogation system has the same capability as a tunable laser source to perform the operational load monitoring and impact damage detection. Though it has a trade-off in the resolution and accuracy, the AWG-based wavelength interrogation system has its unique features. Since an AWG has multiple channels, it has the capability to simultaneously interrogate multiple FBG sensors required by the FBG acoustic sensor system. In addition, an AWG based interrogation system has features of miniaturized size and light weight which makes it ideal for aircraft SHM applications. Recently, we have developed another wavelength interrogation system based on an Echelle diffractive grating (EDG), which is also based on planar lightwave circuits technology and offers the same functions [66] as an AWG. The interrogation system based on this EDG chip is able to achieve the wavelength resolution of less than 1 pm with a measurement accuracy of ±10 pm. We are currently evaluating its performances for both load and acoustic monitoring [67].

## 5. Recommendations and Future Work

There is currently no standard for the FBG acoustic sensor system for aircraft SHM applications. An example specification of FBG sensors on operational load monitoring is proposed in [23]. As a general guidance to apply FBG sensors for damage detections, the system should have the ability to detect: 1–2.5 mm cracks in aluminum sheet, 5 mm cracks in a metallic frame, 100 mm cracks in large areas, and 10% of sheet thickness in corrosion or 15 × 15 mm debonds with at least 90% detect ability. We have recently set up a target performance specification for an FBG acoustic sensor system for aircraft SHM. The proposed sensor system is designed to have the capability of performing both operational load monitoring and damage detection. Part of the specifications is shown as follows:

- Total number of FBG sensors: 32 (potentially up to 64)
- Number of FBG sensors per patch: 8 (7 for strain, 1 for temperature; potentially 16 for strain range of ±2,000 µɛ)
- Number of strain patches per system: 4
- Sensor type: FBG sensors in single mode fiber
- Operational wavelength: centered at 1,550 nm
- Spectral bandwidth per FBG sensor channel: 8 nm
- Strain range per FBG sensor: ±3,500 µɛ
- Damage detection using FBG acoustic sensors: 1–5 mm cracks
- FBG interrogation speed: static to 500 kHz
- FBG sensor reflectivity: more than 70%
- FBG sensor reflection bandwidth: 0.4 nm
- FBG grating length: 5 to 10 mm
- Strain resolution: 1 µɛ
- Temperature resolution: 0.1 °C
- Strain accuracy: 10 µɛ
- Temperature accuracy: 1 °C
- Operating temperature range: −60 to +100 °C

Besides these specifications for the FBG sensors, the packaging and mounting techniques of FBG sensors have also been developed and tested in field applications [15,68,69]. Recent development of laboratory-oriented experiments has identified that FBG sensors are significantly mature for civil applications. However, the FBG sensor technology still requires further development to be effectively employed in the aerospace market, which includes the following:

- Robustness and reliability of the sensor packaging technique
- Sensor locations and mounting layout
- Materials used for mounting sensors on or into structures of different materials
- Establishment of the standards for sensor manufacture

## 6. Conclusions

Requirements for aircraft SHM and an overview of the state-of-the-art fiber optic sensing techniques have been given in this paper. Based on the discussion, FBG sensors were selected to perform the dual functions that an aircraft SHM system should have in the future, which were operational load monitoring and damage detection. A review of the FBG acoustic sensor system was provided, including the principle, technique development and its wavelength interrogation system. Finally, a specification of an FBG acoustic sensor system with its targeted performance for aircraft SHM was listed based on our understanding and perspectives.

## Figures and Tables

**Table 1.** Evaluation of selected fiber optic sensor technologies.

	**Fabry-Perot Interferometric Sensors**	**SOFO Interferometric Sensors**	OTDR^*^	ROTDR^*^	BOTDR^*^	**Fiber Bragg Grating Sensors**
**Sensor type**	Point	Long gauge	Distributed	Distributed	Distributed	-Point-Semi-distributed
**Main sensing parameters**	-Temperature -Strain -Rotation -Pressure	-Deformation -Strain -Force	-Fiber loss -Break location	-Temperature	-Temperature -Strain	-Temperature -Strain -Rotation -Pressure
**Multiplexing**	-Parallel -Time-division	-Parallel -Time-division	Distributed	Distributed	Distributed	-Quasi distributed -Wavelength-division
**Measurement point in one line**	1	1	Depending on the range and resolution	Depending on the range and resolution	Depending on the range and resolution	10–50
**Typical resolution Strain (μStrain) Temperature (°C)**	0.15 0.1	1 N/A	N/A 0N/A	N/A 0.1	20 0.2	1 0.1
**Capability for large wavelength shift detection (∼10 nm)**	Yes	No	No	No	No	Yes
**Spatial resolution**	0.1	0.1	1–10	1	1	0.1
**Capability of fast response for acoustic signal detection (>100 kHz)**	Yes	No	No	No	No	Yes
**Advantages**	-High sensitivity -Accurate	-Long gauge -High spatial rsolution	Wide applications	-Infinite sensing points -Fiber integrated	-Infinite sensing points -Fiber integrated	-Linearity in response -Accurate -High resolution -Inherent WDM encoding
**Disadvantages**	Single point	Low speed (10 s)	Detection limitations	-Temperature only -High cost	Cross sensitivity	Cross sensitivity

* Refer to Section 3.2
